# Methyl 2-(3-oxo-3,4-dihydro-2*H*-1,4-benzothia­zin-4-yl)acetate

**DOI:** 10.1107/S1600536811006477

**Published:** 2011-02-26

**Authors:** Yamna Barryala, Stéphane Massip, Saïd Lazar, El Mokhtar Essassi, Hafid Zouihri

**Affiliations:** a Laboratoire de Chimie Organique et Etudes Physico-chimiques ENS Takaddoum, Rabat, Morocco; b Laboratoire de Chimie Physique et Minérale, Service de Cristallographie, Université Victor Ségalen Bordeaux 2, Bordeaux Cedex, France; c Laboratoire de Biochimie, Environnement et Agroalimentaire (URAC 36), Faculté des Sciences et Techniques Mohammedia, Université Hassan II Mohammedia–Casablana, BP 146, 20800 Mohammedia, Morocco; d Laboratoire de Chimie Organique Hétérocyclique, Faculté des Sciences de Rabat, Morocco; e Laboratoires de Diffraction des Rayons X, Centre Nationale pour la Recherche Scientifique et Technique, Rabat, Morocco

## Abstract

In the crystal structure of the title compound, C_11_H_11_NO_3_S, the mol­ecules are linked by inter­molecular C—H⋯O hydrogen-bond inter­actions. The heterocyclic thia­zine ring adopts a conformation inter­mediate between twist and boat.

## Related literature

For general background to the synthesis of benzothia­zines, see: Harmata *et al.* (2005 ▶). For the pharmacological activity of benzothia­zine derivatives, see: Lopatina *et al.* (1982 ▶). For related structures, see: Saeed *et al.* (2010 ▶); Aouine *et al.* (2010 ▶).
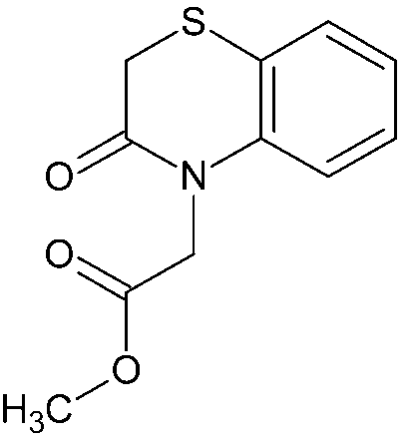


## Experimental

### 

#### Crystal data

C_11_H_11_NO_3_S
*M* *_r_* = 237.27
Monoclinic, 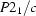

*a* = 17.347 (5) Å
*b* = 8.724 (2) Å
*c* = 7.274 (1) Å
β = 98.71 (2)°
*V* = 1088.1 (4) Å^3^
*Z* = 4
Cu *K*α radiation
μ = 2.59 mm^−1^
*T* = 296 K
0.20 × 0.15 × 0.15 mm

#### Data collection

Enraf–Nonius CAD-4 diffractometer
Absorption correction: ψ scan (North *et al.*, 1968 ▶) *T*_min_ = 0.625, *T*_max_ = 0.697
1852 measured reflections
1852 independent reflections
1654 reflections with *I* > 2σ(*I*)
2 standard reflections every 90 min  intensity decay: none

#### Refinement

*R*[*F* ^2^ > 2σ(*F* ^2^)] = 0.043
*wR*(*F* ^2^) = 0.123
*S* = 1.03
1852 reflections
147 parameters
H-atom parameters constrained
Δρ_max_ = 0.31 e Å^−3^
Δρ_min_ = −0.28 e Å^−3^

### 

Data collection: *CAD-4 Software* (Enraf–Nonius, 1989 ▶); cell refinement: *CAD-4 Software*; data reduction: *CAD-4 Software*; program(s) used to solve structure: *SHELXS97* (Sheldrick, 2008 ▶); program(s) used to refine structure: *SHELXL97* (Sheldrick, 2008 ▶); molecular graphics: *PLATON* (Spek, 2009 ▶); software used to prepare material for publication: *publCIF* (Westrip, 2010 ▶).

## Supplementary Material

Crystal structure: contains datablocks I, global. DOI: 10.1107/S1600536811006477/bt5472sup1.cif

Structure factors: contains datablocks I. DOI: 10.1107/S1600536811006477/bt5472Isup2.hkl

Additional supplementary materials:  crystallographic information; 3D view; checkCIF report

## Figures and Tables

**Table 1:** Hydrogen-bond geometry (Å, °)

*D*—H⋯*A*	*D*—H	H⋯*A*	*D*⋯*A*	*D*—H⋯*A*
C4—H4⋯O14	0.93	2.51	3.411 (3)	164

**Fig. 1.**
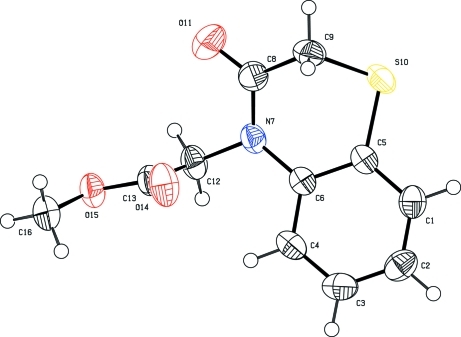
Molecular view of the title compound showing the atom-labeling scheme. Displacement ellipsoids are drawn at the 50% probability level. H atoms are represented as small spheres of arbitrary radii.

**Fig. 2.**
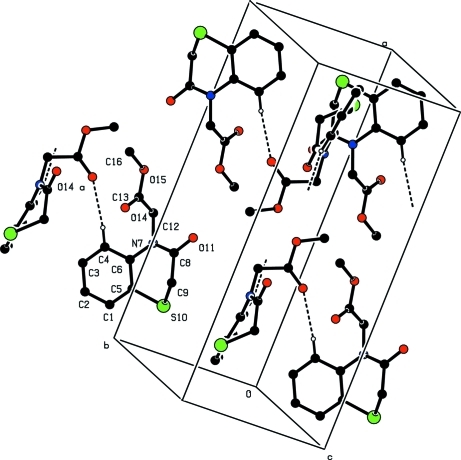
Partial packing view showing the chain formed by C—H···O hydrogen bondings. H atoms not involved in hydrogen bonds have been omitted for clarity.

## supplementary crystallographic information

### Comment

Several derivatives of benzothiazines, particularly those carrying a keto group on the thiazine part of the molecule, show stimulant and antidepressant activity. [Lopatina *et al.* 1982].

The goal of the present work was the synthesis, the crystal structure determination and the biological teste of the methyl (3-oxo-2,3-dihydro-4*H*-1,4-benzothiazin-4-yl)acetate. [Harmata *et al.* 2005].

In the crystal structure of the title compound (Fig. 1), the molecules exhibit C···H—O intermolecular H-bonds interactions (Fig. 2). The heterocyclic thiazine ring adopt half-chair conformation with the S and N atoms displaced by 0.357 (5) and 0.304 (15) Å, respectively, on the opposite sides from the mean plane formed by the remaining ring atoms. The methyl acetate group, which is almost planar with the r. m.s deviaton of 0.042 (14) Å, is iclined at dihedral angle of 88.31 (9)° and 74.67 (9)° with respect to the thiazine and benzene ring respectively.

The dihedral angle between the aromatic benzene ring C1–C6 and thiazine ring C5/C6/N7/C8/C9/S10 is 17.02 (9)° while the methyl acetate group C12/C13/O14/O15/C16 is oriented at dihedral angle of 81.30 (8)° with respect to the benzothiazine ring.

In the title compound (Fig. 1), the bond distances and angles agree with the cortresponding bond distances and angles reported in related compounds [Saeed *et al.* 2010 and Aouine *et al.* 2010].

### Experimental

To 1,4-benzithiazin-3-one (0.25 g, 1.5 mmol), potassium carbonate (0.41 g, 3 mmol), in ketone (15 ml) was added methyl chloroacetate (0.32 g, 3 mmol). The mixture was heated to reflux for 48 h. The salts were removed by filtration and the filtrate concentrated under reduced pressure. The residue was separated by chromatography on a column of silica gel with dichloromethane/diethyl ether (9/1) as eluent. Crystals were isolated when the solvent was allowed to evaporate.

### Refinement

All H atoms were fixed geometrically and treated as riding with C—H = 0.97 Å (methyne) and 0.93Å (aromatic) with U iso (H) = 1.2Ueq(C).

### Crystal data

**Table:**

C_11_H_11_NO_3_S	*F*(000) = 496
*M**_r_* = 237.27	*D*_x_ = 1.448 Mg m^−^^3^
Monoclinic, *P*2_1_/*c*	Cu *K*α radiation, λ = 1.54180 Å
Hall symbol: -P 2ybc	Cell parameters from 25 reflections
*a* = 17.347 (5) Å	θ = 25.0–35.0°
*b* = 8.724 (2) Å	µ = 2.59 mm^−^^1^
*c* = 7.274 (1) Å	*T* = 296 K
β = 98.71 (2)°	Prism, colourless
*V* = 1088.1 (4) Å^3^	0.20 × 0.15 × 0.15 mm
*Z* = 4	

### Data collection

**Table:**

Enraf–Nonius CAD-4 diffractometer	1654 reflections with *I* > 2σ(*I*)
Radiation source: fine-focus sealed tube	*R*_int_ = 0.000
graphite	θ_max_ = 64.9°, θ_min_ = 2.6°
ω–2θ scans	*h* = −20→20
Absorption correction: ψ scan (North *et al.*, 1968)	*k* = 0→10
*T*_min_ = 0.625, *T*_max_ = 0.697	*l* = 0→8
1852 measured reflections	2 standard reflections every 90 min
1852 independent reflections	intensity decay: none

### Refinement

**Table:**

Refinement on *F*^2^	Secondary atom site location: difference Fourier map
Least-squares matrix: full	Hydrogen site location: inferred from neighbouring sites
*R*[*F*^2^ > 2σ(*F*^2^)] = 0.043	H-atom parameters constrained
*wR*(*F*^2^) = 0.123	*w* = 1/[σ^2^(*F*_o_^2^) + (0.0891*P*)^2^ + 0.2952*P*] where *P* = (*F*_o_^2^ + 2*F*_c_^2^)/3
*S* = 1.03	(Δ/σ)_max_ < 0.001
1852 reflections	Δρ_max_ = 0.31 e Å^−^^3^
147 parameters	Δρ_min_ = −0.28 e Å^−^^3^
0 restraints	Extinction correction: *SHELXL97* (Sheldrick, 2008), Fc^*^=kFc[1+0.001xFc^2^λ^3^/sin(2θ)]^-1/4^
Primary atom site location: structure-invariant direct methods	Extinction coefficient: 0.025 (2)

### Special details

**Table:**

Geometry. All e.s.d.'s (except the e.s.d. in the dihedral angle between two l.s. planes) are estimated using the full covariance matrix. The cell e.s.d.'s are taken into account individually in the estimation of e.s.d.'s in distances, angles and torsion angles; correlations between e.s.d.'s in cell parameters are only used when they are defined by crystal symmetry. An approximate (isotropic) treatment of cell e.s.d.'s is used for estimating e.s.d.'s involving l.s. planes.
Refinement. Refinement of *F*^2^ against ALL reflections. The weighted *R*-factor *wR* and goodness of fit *S* are based on *F*^2^, conventional *R*-factors *R* are based on *F*, with *F* set to zero for negative *F*^2^. The threshold expression of *F*^2^ > σ(*F*^2^) is used only for calculating *R*-factors(gt) *etc*. and is not relevant to the choice of reflections for refinement. *R*-factors based on *F*^2^ are statistically about twice as large as those based on *F*, and *R*- factors based on ALL data will be even larger.

### Fractional atomic coordinates and isotropic or equivalent isotropic displacement parameters (Å^2^)

**Table:**

	*x*	*y*	*z*	*U*_iso_*/*U*_eq_	
C1	0.06412 (11)	1.0010 (2)	−0.1876 (3)	0.0408 (5)	
C12	0.34845 (11)	0.9981 (2)	−0.1202 (3)	0.0425 (5)	
C13	0.38184 (11)	1.1551 (2)	−0.0708 (3)	0.0385 (5)	
C16	0.48971 (13)	1.3152 (3)	−0.0896 (3)	0.0523 (6)	
C2	0.06020 (13)	1.0995 (2)	−0.3366 (3)	0.0462 (5)	
C3	0.12837 (14)	1.1575 (2)	−0.3843 (3)	0.0477 (5)	
C4	0.19978 (12)	1.1181 (2)	−0.2852 (3)	0.0411 (5)	
C5	0.13542 (10)	0.9596 (2)	−0.0860 (2)	0.0342 (4)	
C6	0.20474 (10)	1.0183 (2)	−0.1350 (2)	0.0325 (4)	
C8	0.29001 (11)	0.9244 (2)	0.1449 (3)	0.0424 (5)	
C9	0.22014 (12)	0.9212 (3)	0.2432 (3)	0.0480 (5)	
H1	0.0183	0.9615	−0.1545	0.049*	
H12A	0.3363	0.9889	−0.2544	0.051*	
H12B	0.3870	0.9207	−0.0761	0.051*	
H16A	0.4570	1.3905	−0.1589	0.078*	
H16B	0.5394	1.3128	−0.1325	0.078*	
H16C	0.4970	1.3413	0.0401	0.078*	
H2	0.0122	1.1265	−0.4041	0.055*	
H3	0.1262	1.2242	−0.4847	0.057*	
H4	0.2451	1.1588	−0.3192	0.049*	
H9A	0.2064	1.0251	0.2730	0.058*	
H9B	0.2327	0.8649	0.3589	0.058*	
N7	0.27801 (9)	0.97159 (18)	−0.0378 (2)	0.0371 (4)	
O11	0.35438 (9)	0.8896 (2)	0.2240 (2)	0.0645 (5)	
O14	0.34929 (8)	1.25486 (18)	−0.0003 (2)	0.0561 (5)	
O15	0.45320 (8)	1.16608 (16)	−0.1160 (2)	0.0454 (4)	
S10	0.13827 (3)	0.83215 (6)	0.10137 (7)	0.0470 (3)	

### Atomic displacement parameters (Å^2^)

**Table:**

	*U*^11^	*U*^22^	*U*^33^	*U*^12^	*U*^13^	*U*^23^
C1	0.0361 (10)	0.0407 (10)	0.0462 (11)	−0.0021 (8)	0.0082 (8)	−0.0077 (8)
C2	0.0487 (11)	0.0415 (11)	0.0450 (11)	0.0044 (9)	−0.0043 (9)	−0.0055 (9)
C3	0.0651 (14)	0.0391 (11)	0.0370 (10)	−0.0035 (9)	0.0012 (9)	0.0033 (8)
C4	0.0502 (11)	0.0370 (10)	0.0385 (10)	−0.0082 (9)	0.0144 (8)	0.0011 (8)
C5	0.0378 (9)	0.0294 (9)	0.0373 (10)	−0.0026 (7)	0.0121 (7)	−0.0040 (7)
C6	0.0359 (9)	0.0287 (9)	0.0343 (9)	−0.0022 (7)	0.0098 (7)	−0.0042 (7)
N7	0.0312 (8)	0.0376 (8)	0.0447 (9)	−0.0005 (6)	0.0128 (6)	0.0019 (7)
C8	0.0408 (10)	0.0393 (10)	0.0471 (11)	0.0024 (8)	0.0071 (8)	0.0026 (8)
C9	0.0509 (11)	0.0567 (13)	0.0379 (10)	0.0024 (10)	0.0116 (9)	0.0121 (9)
S10	0.0442 (4)	0.0470 (4)	0.0529 (4)	−0.0048 (2)	0.0170 (2)	0.0152 (2)
O11	0.0449 (9)	0.0783 (12)	0.0667 (11)	0.0108 (8)	−0.0030 (7)	0.0096 (9)
C12	0.0334 (9)	0.0418 (11)	0.0560 (12)	0.0009 (8)	0.0185 (8)	−0.0028 (9)
C13	0.0295 (9)	0.0454 (11)	0.0419 (10)	−0.0017 (8)	0.0098 (7)	−0.0010 (8)
O14	0.0455 (9)	0.0543 (10)	0.0735 (11)	−0.0063 (7)	0.0257 (8)	−0.0220 (8)
O15	0.0317 (7)	0.0491 (8)	0.0579 (9)	−0.0035 (5)	0.0147 (6)	0.0000 (6)
C16	0.0405 (11)	0.0562 (13)	0.0605 (13)	−0.0111 (9)	0.0085 (10)	0.0037 (11)

### Geometric parameters (Å, °)

**Table:**

S10—C5	1.7538 (17)	C5—C6	1.402 (2)
S10—C9	1.800 (2)	C8—C9	1.498 (3)
O11—C8	1.215 (3)	C12—C13	1.509 (3)
O14—C13	1.195 (2)	C1—H1	0.9300
O15—C13	1.332 (2)	C2—H2	0.9300
O15—C16	1.447 (3)	C3—H3	0.9300
N7—C6	1.418 (2)	C4—H4	0.9300
N7—C8	1.377 (3)	C9—H9A	0.9700
N7—C12	1.459 (3)	C9—H9B	0.9700
C1—C2	1.377 (3)	C12—H12A	0.9700
C1—C5	1.389 (3)	C12—H12B	0.9700
C2—C3	1.378 (3)	C16—H16A	0.9600
C3—C4	1.379 (3)	C16—H16B	0.9600
C4—C6	1.390 (3)	C16—H16C	0.9600
			
C5—S10—C9	95.67 (10)	C5—C1—H1	120.00
C13—O15—C16	115.88 (16)	C1—C2—H2	120.00
C6—N7—C8	124.10 (15)	C3—C2—H2	121.00
C6—N7—C12	119.55 (15)	C2—C3—H3	120.00
C8—N7—C12	115.50 (16)	C4—C3—H3	120.00
C2—C1—C5	121.00 (18)	C3—C4—H4	120.00
C1—C2—C3	119.0 (2)	C6—C4—H4	120.00
C2—C3—C4	120.93 (19)	S10—C9—H9A	109.00
C3—C4—C6	120.73 (19)	S10—C9—H9B	109.00
S10—C5—C1	119.76 (14)	C8—C9—H9A	109.00
S10—C5—C6	120.32 (13)	C8—C9—H9B	109.00
C1—C5—C6	119.92 (15)	H9A—C9—H9B	108.00
N7—C6—C4	121.09 (16)	N7—C12—H12A	109.00
N7—C6—C5	120.47 (14)	N7—C12—H12B	109.00
C4—C6—C5	118.39 (16)	C13—C12—H12A	109.00
O11—C8—N7	121.72 (18)	C13—C12—H12B	109.00
O11—C8—C9	121.43 (19)	H12A—C12—H12B	108.00
N7—C8—C9	116.83 (17)	O15—C16—H16A	109.00
S10—C9—C8	111.08 (15)	O15—C16—H16B	109.00
N7—C12—C13	111.18 (16)	O15—C16—H16C	109.00
O14—C13—O15	124.83 (17)	H16A—C16—H16B	109.00
O14—C13—C12	125.04 (18)	H16A—C16—H16C	109.00
O15—C13—C12	110.13 (16)	H16B—C16—H16C	109.00
C2—C1—H1	119.00		

### Hydrogen-bond geometry (Å, °)

**Table:**

*D*—H···*A*	*D*—H	H···*A*	*D*···*A*	*D*—H···*A*
C4—H4···O14^i^	0.93	2.51	3.411 (3)	164

Symmetry codes: (i) .
